# Effective Utilisation of Halophyte Biomass from Saline Soils for Biorefinering Processes

**DOI:** 10.3390/molecules26175393

**Published:** 2021-09-05

**Authors:** Jolanta Batog, Krzysztof Bujnowicz, Weronika Gieparda, Aleksandra Wawro, Szymon Rojewski

**Affiliations:** Institute of Natural Fibres and Medicinal Plants—National Research Institute, Wojska Polskiego 71B, 60-630 Poznan, Poland; jolanta.batog@iwnirz.pl (J.B.); krzysztof.bujnowicz@iwnirz.pl (K.B.); weronika.gieparda@iwnirz.pl (W.G.); szymon.rojewski@iwnirz.pl (S.R.)

**Keywords:** halophyte biomass, saline soils, pretreatment, SSF, biorefinering process, bioethanol, biocomposites

## Abstract

The salinity of European soil is increasing every year, causing severe economic damage (estimated 1–3 million hectares in the enlarged EU). This study uses the biomass of halophytes—tall fescue (grass) and hemp of the Białobrzeskie variety from saline soils—for bioenergy, second generation biofuels and designing new materials—fillers for polymer composites. In the bioethanol obtaining process, in the first stage, the grass and hemp biomass were pretreated with 1.5% NaOH. Before and after the treatment, the chemical composition was determined and the FTIR spectra and SEM pictures were taken. Then, the process of simultaneous saccharification and fermentation (SSF) was carried out. The concentration of ethanol for both the grass and hemp biomass was approx. 7 g·L^−1^ (14 g·100 g^−1^ of raw material). In addition, trials of obtaining green composites with halophyte biomass using polymers (PP) and biopolymers (PLA) as a matrix were performed. The mechanical properties of the composites (tensile and flexural tests) were determined. It was found that the addition of a compatibilizer improved the adhesion at the interface of PP composites with a hemp filler. In conclusion, the grass and hemp biomass were found to be an interesting and promising source to be used for bioethanol and biocomposites production. The use of annually renewable plant biomass from saline soils for biorefinering processes opens up opportunities for the development of a new value chains and new approaches to sustainable agriculture.

## 1. Introduction

The dynamic development of economic activities often causes changes in the environment and human life quality. The world population is anticipated to grow 40% within 40–50 years with unprecedented demands for energy, food, freshwater and a clean environment. At 43% of the total landmass, exploiting the Earth’s arid and semi-arid lands becomes a matter of necessity [1].

Soil salinisation effects are seen as a major cause of desertification and, therefore, are a serious form of soil degradation, endangering the potential use of European soils. The contamination of the environment by the excessive salinity of soils may be induced during coastal flooding, by the intensive agriculture practices or under the pressure of climate changes (aridisation phenomena). Such soils have lower or zero suitability for traditional agriculture production, influencing the decline of regional economics [2].

The remediation of these sites or the implementation of new ways to use them are necessary to achieve the restoration of agricultural production and thus economic growth. This can be met by the use of halophytes in the development of an integrated system of soil bioremediation and biomass biorefinery [3].

Tall fescue (*Festuca arundinacea*) and hemp (*Cannabis sativa* L.) are characterised by high biomass production and wide ecological amplitude, including tolerance to soil salinity.

Tall fescue creates large, dense and deeply rooted clumps, showing considerable tolerance to unfavourable habitat conditions. It occurs on roadsides, meadows (including saline meadows) and alluvia.

Hemp is a plant with a short growing season, resistant to diseases and unfavourable environmental conditions, with a dry matter yield of 10–15 t·ha^−1^. They improve soil quality and are useful for the restoration of brownfield sites; 1 ha of hemp binds about 11 t of CO_2_ [4,5,6].

This study addresses the challenge of improving the knowledge and understanding of halophyte species in order to better use the biomass from these plants for the production of new environmentally friendly materials [7].

EU Member States have been obliged to achieve a certain share of biofuels in transport and to take measures to reduce greenhouse gas emissions. According to the EU RED II Directive, the contribution of advanced biofuels and biogas produced, among others, from lignocellulosic raw materials, as a share of final energy consumption in the transport sector, it is expected to amount to at least 0.2% by 2022, 1% by 2025 and 3.5% by 2030 [8]. Therefore, it is very interesting and important to try to obtain bioethanol from the halophyte biomass from saline soils [3].

The production of lignocellulosic ethanol involves the breakdown of cell walls into individual polymers and the hydrolysis of carbohydrates into simple sugars. Plant biomass contains the lignocellulose complex (cellulose, hemicellulose, lignin), which is relatively resistant to biodegradation. The process of converting halophyte biomass to bioethanol involves several steps, from the preparation of the plant material (physical and chemical treatment), through enzymatic hydrolysis, to ethanol fermentation. The purpose of the pretreatment of biomass is to crush the solid phase and loosen the compact structure of the lignocellulose. Moreover, the method combining cellulose hydrolysis with sugar fermentation in one bioreactor (SSF process), where enzymes must be adapted to the conditions of the fermentation process (30–40 °C), seems to be effective and economical [9,10,11].

Another important point is that around 25 million tons of plastic waste are produced annually in the EU. The market is dominated by products made from materials such as PE, PP, PET, PS and PVC, that take several hundred years to decompose. More and more emphasis is placed on the need to significantly reduce the amount of plastic waste (Single-Use Plastics Directive) [12,13].

The plastics processing industry is looking for material innovations that meet customer expectations and legal requirements; therefore, in the next few years, an increased interest in environmentally friendly biodegradable materials is expected. An example is the production of polylactic acid (PLA), which is one of the leading bioplastics on the market [14,15,16].

The barrier to the wider use of biodegradable plastics is the high production costs, higher than traditionally used plastics.

A method to improve the economics may be the use of natural fillers from the biomass of annual plants (flax and hemp biomass), which are cheaper than biodegradable plastics [17,18]. Materials composed of thermoplastic polymers with natural fillers have functional properties that do not differ from pure polymers and may be used in various fields of the economy, including the automotive industry, transport, construction and the furniture industry.

Due to the expected increase in the production of biocomposite materials in the EU in the coming years, an increase in the demand for biocomponents based on natural resources, including natural fibres, should be expected.

Moreover, to fully understand the impacts of biorefineries, taking a comprehensive view of sustainability, it is necessary to consider a wider range of factors including economics and social and environmental issues. The potential benefits of strengthening the halophyte based biorefineries are certainly reduced greenhouse gas emissions, less dependence on fossil resources, better land and natural resource management and improved food security and soil quality. In addition, a significant positive effect of biobased industries is the creation of employment opportunities in rural areas, as well as the possibility of creating new markets for agriculture (biofuels, green composites) in synergy with the existing ones.

The aim of the presented study is to indicate the possibility of using tall fescue and hemp from saline soils in the process of obtaining second generation biofuels and for the design of green biocomposites. Thus far, only a few reports of the use of halophyte biomass from degraded areas in biorefining processes have been published [3,19,20]. A novelty in this manuscript is an indication of the possibility of managing degraded, saline soils through the production of biomass for industrial purposes, including bioenergy, which is in line with the idea of sustainable development, that is now a priority policy of the European Union.

## 2. Results and Discussion

### 2.1. Bioethanol Production Process

#### 2.1.1. Halophyte Biomass Pretreatment

The biomass crushed on a knife mill with a mesh of 2 mm was treated with alkaline. One of the most popular alkaline reagents used in the treatment of raw plant material is sodium hydroxide. The main purpose of the pretreatment of lignocellulosic biomass is to crush the solid phase and loosen the compact lignocellulose structure. After a chemical pretreatment test with sodium hydroxide (1.5–3%, 90 °C), sulfuric acid (1–2%, 121 °C) and hot water (135 °C), it turned out that the most effective treatment was alkaline with 1.5% NaOH. Table 1 shows the alkaline treatment of the grass and hemp biomass from saline soils using 1.5% NaOH.

It was found that grass from the saline soil was characterised by an almost two times higher content of reducing sugars than hemp. The results indicate that grass is more susceptible to alkaline treatment than hemp.

An effective chemical treatment should ensure the destruction of the crystalline structure of cellulose, the separation of lignin from carbohydrates and, thus, an increase in the availability of the substrate for further biofuel production processes [21].

To confirm the efficiency of the alkaline treatment, a determination of the chemical composition of the halophyte biomass after the NaOH treatment was performed and compared to the chemical composition of the biomass before the pretreatment. The results are presented in Table 2.

The analysis of the chemical composition of the halophyte biomass before and after the treatment showed that the alkaline effect caused a visible increase in the cellulose content by over 10%, especially for grass, which saw a 17% increase. In addition, a partial degradation of hemicellulose was found (as much as 11% for hemp). In the case of the lignin content for the hemp biomass, an increase was observed after the alkaline pretreatment (over 3%), and in the case of grass—an almost 5% reduction. Similar observations regarding the hemp biomass were presented by Stevulova et al. [22], who showed that the lignin content after pretreatment with sodium hydroxide was 7% higher than before.

It was found that in the case of the grass biomass, both the higher cellulose growth and the reduction in lignin content positively influenced the higher values of the reducing sugars released after the pretreatment with sodium hydroxide compared to the hemp biomass (see Table 1). It should be emphasised that one of the main goals of the chemical treatment of lignocellulosic materials is the removal of lignin, which is a strong obstacle in the process of biomass conversion.

**Figure 1:**
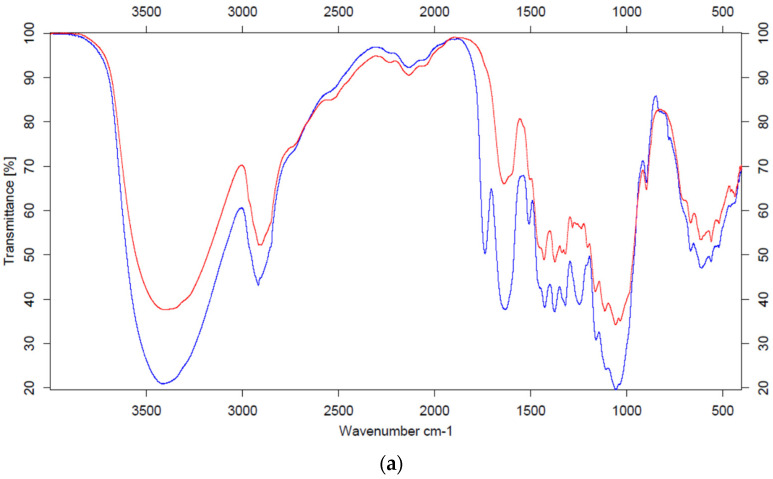

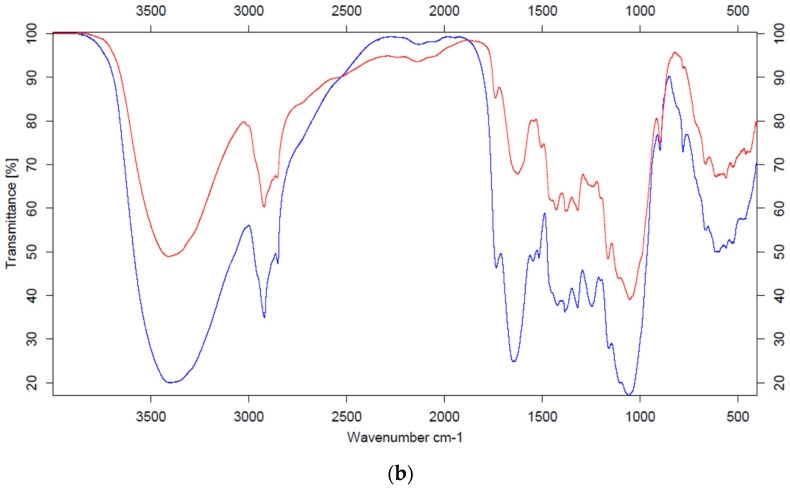
(**a**) FTIR spectra of hemp biomass from saline soil before (blue) and after (red) NaOH pretreatment. (**b**) FTIR spectra of grass biomass from saline soil before (blue) and after (red) NaOH pretreatment.

The effect of alkaline treatment on the grass and hemp biomass from saline soils was confirmed using Fourier Transform Infrared Spectrometer (FTIR) between 600–4000 cm^−1^ shown in the Figure 1a,b and using Scanning Electron Microscopy (SEM).

The broad band, ranging from about 3500 to 3000 cm^−1^, comes from the O–H stretching vibrations in the cellulose molecule. In all the cases, the band after pretreatment is less intense and narrower. The band at 2900 cm^–1^, resulting from the stretching vibrations of the C–H group (cellulose), is also less intense after pretreatment. The decrease in the intensity of these bands, despite the increase in the cellulose content after treatment (confirmed by results from the chemical composition), may be caused by the reduction in the crystalline structure of cellulose [23,24]. These bands, in the case of different materials, have a different shape (for grass, two characteristic peaks appear in this place, while for hemp, one broad band is visible). The vibration band visible at 1730 cm^−1^, resulting from the C=O stretching vibrations of the acetyl group in hemicellulose [25], disappears in the case of hemp after treatment, while in the case of grass, it is significantly reduced. This is due to the degradation of hemicellulose during the alkaline pretreatment of biomass. In turn, the lower intensity of the band at 1600 cm^−1^ corresponding to the O–H stretching vibrations, reflecting the amount of water absorbed in the sample, is probably caused by the loss of water during the drying process of the samples after pretreatment [26]. The absorption band at 1510 cm^−1^, resulting from the vibrations of the aromatic ring in lignin in the case of grass, decreases after alkaline treatment, which is reflected in the chemical composition of the biomass. Significant changes are visible in the band at 1230 cm^−1^. This band is attributed to the vibration of the guaiacyl ring in lignin, as well as to the vibration of the C–O groups in pectin. After the alkaline treatment, this band disappears completely or significantly loses its intensity, which may indicate a reduction in both lignin and pectin. Going to the lower wavenumber values, the following three more characteristic bands for the cellulose molecule appear: 1160 cm^−1^ (asymmetric C–O–C stretching vibrations), 1110 cm^−1^ (C–OH skeletal vibrations) and 1050 cm^−1^ (C–O–C skeletal vibrations of the pyranose ring) [27]. These bands, despite the increase in cellulose content after chemical treatment, are reduced. This is characteristic of each biomass tested and can be attributed to the reduction in the crystalline structure of the cellulose after treatment.

**Figure 2:**
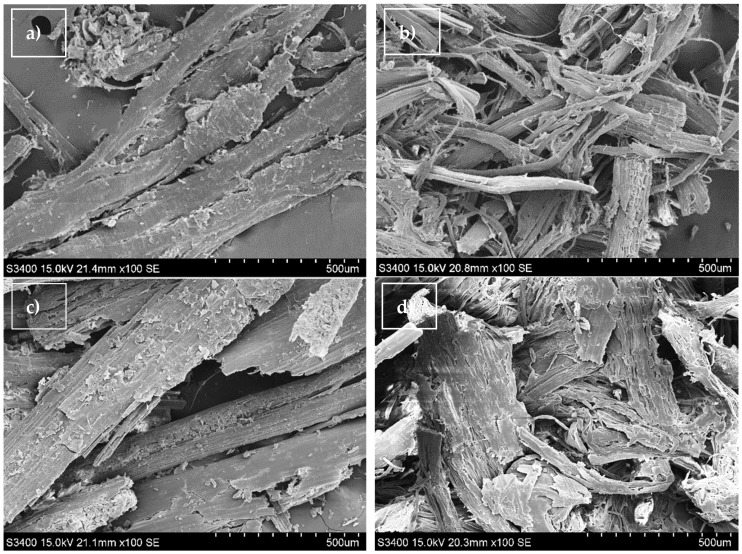
SEM images of halophyte biomass: (**a**) hemp biomass before NaOH pretreatment, (**b**) hemp biomass after NaOH pretreatment, (**c**) grass biomass before NaOH pretreatment, (**d**) grass biomass after NaOH pretreatment.

Significant changes on the surface of the grass and hemp biomass were observed and presented in the SEM images taken before and after pretreatment (Figure 2).

Figure 2a,c show that the untreated grass and hemp biomass have intact and rigid structures with well-ordered fibrous skeletons, effectively blocking access to lignocellulose [28]. After the pretreatment of the grass and hemp biomass with sodium hydroxide for, similar changes were observed on the surface of the biomass. The SEM images of the halophyte biomass after alkaline treatment (Figure 2b,d) show damage to the structure of the biomass and the appearance of hollow areas, which increases its surface and positively affects the enzymatic availability and digestibility of the biomass [29,30].

To sum up, the main goal of the chemical treatment of lignocellulosic materials for biofuel is to increase the availability of the biomass structure by the decrystallisation of cellulose and the removal of lignin.

#### 2.1.2. Enzyme Complex

The second stage of the process of obtaining bioethanol from plant biomass is the enzymatic hydrolysis process. The breakdown of cellulose into simple sugars requires the synergistic action of cellulases—endoglucanases, cellobiohydrolases and ß-glucosidases. Two enzyme preparations were selected for the research—Flashzyme Plus 200 and Celluclast 1.5 L [31]. In order to select the enzyme complex for the SSF process, tests were performed using selected enzymes and their supplementation with glucosidase and xylanase (Table 3).

For the hydrolysis of the solid fraction in the SSF process, the Flashzyme Plus 200/Celluclast 1.5 L complex in the proportion of 50/50 was selected for the hemp biomass, and the Flashzyme Plus 200/Celluclast 1.5 L complex in the proportion of 70/30 for the grass biomass. On the basis of the enzymatic test, it was found that the content of the released reducing sugars for the grass biomass (892 mg·g^−1^) was two times higher than for the hemp biomass (430 mg·g^−1^).

#### 2.1.3. Simultaneous Saccharification and Fermentation (SSF)

**Figure 3:**
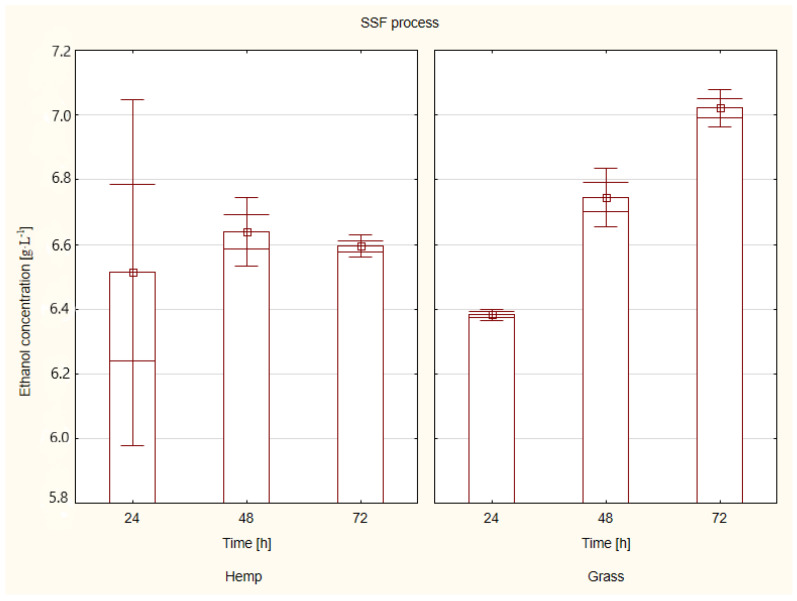
Ethanol concentration of hemp and grass biomass after the SSF process (Statistica 13.0).

The SSF process, consisting of simultaneous hydrolysis and fermentation, takes place under conditions ensuring the optimal synergy of enzymes and distillery yeast. After carrying out the fermentation tests with selected parameters, the amount of ethanol was determined (HPLC). Figure 3 shows the ethanol concentration after the SSF process for the hemp and grass biomass from saline soils.

It was noted that at 48 h, there was a slight decrease in the ethanol concentration for the hemp biomass but an increase for the grass biomass. The highest ethanol concentration for the hemp biomass was observed at 48 h and it was 6.6 g·L^−1^, which is 13.3 g·100 g^−1^ of raw material. In turn, for the grass biomass, the highest ethanol concentration, equal to 7.0 g·L^−1^ (14.0 g·100 g^−1^ of raw material), was recorded after 72 h.

According to the literature reports, SSF is a beneficial process due to its short processing time, small reactor volume and high ethanol yield, as bioethanol is produced immediately with glucose conversion [32].

Taufikurahman and Sherly [33] conducted similar research on the production of bioethanol from the biomass of Napier grass. After 96 h of enzymatic hydrolysis and fermentation, they obtained an ethanol concentration of 1.25 g·L^−1^. In turn, Riadi et al. [34] conducted research on lignocellulosic biomass in the form of sugarcane bagasse. They first optimised the alkaline pretreatment, followed by enzymatic hydrolysis and ethanol fermentation. After the 48-h fermentation process, they obtained an ethanol concentration of 5.84 g·L^−1^. Obtaining bioethanol from hemp biomass was carried out by Orlygsson [35] and after the pretreatment with 0.5% NaOH and SSF process, the ethanol concentration was approx. 1 g·L^−1^.

Summing up, it should be emphasised that both the hemp and grass biomass obtained from crops on saline soils have great potential in the process of obtaining bioethanol.

### 2.2. Biocomposite Production Process

#### 2.2.1. Fillers from Halophyte Biomass

Natural fillers with particles less than 1 mm were obtained from the grinding of the halophyte biomass. It was assumed that the fraction below 1 mm would be a compromise, allowing for the effective processing of the obtained biocomposites in the injection moulding process, taking into account the diameters of the most commonly used injection nozzles, the simplification of the biomass grinding process and the lower costs of preparing natural fillers. The sieve analysis of the natural fillers was performed, and their humidity was determined. The detailed share of the individual fractions is presented in Table 4.

The bulk density of the hemp fillers (0.19 g·mL^−1^) and the grass fillers (0.22 g·mL^−1^) were determined.

#### 2.2.2. Mechanical Properties of Biocomposites

**Figure 4:**
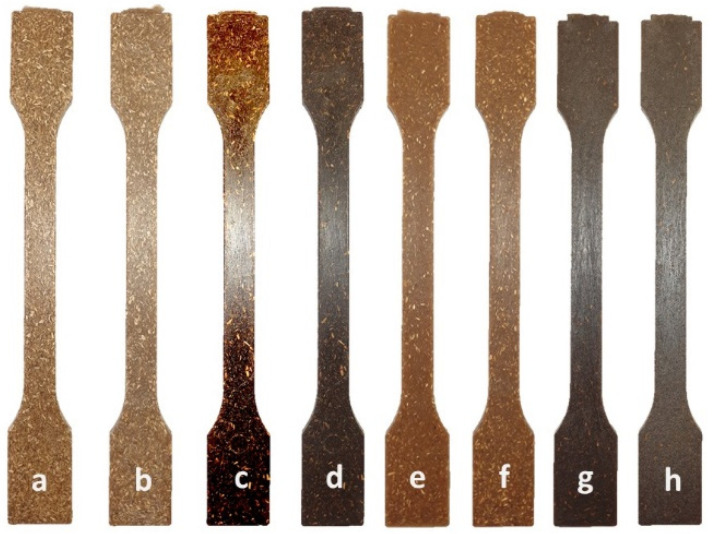
Test specimens of composites and biocomposites with halophyte biomass: (**a**) PP/Hemp20, (**b**) PP/Hemp30, (**c**) PP/Grass20, (**d**) PP/Grass30, (**e**) PLA/Hemp20, (**f**) PLA/Hemp30, (**g**) PLA/Grass20, (**h**) PLA/Grass30.

The multipurpose test specimens—type A in accordance with ISO 3167 [36], used to determine the mechanical properties of composites and biocomposites with halophyte biomass—are shown in Figure 4.

Table 5 and Table 6 show the effect of the different fillers from halophyte biomass on both the tensile and flexural properties of the polypropylene composites.

The 24–38% reduction in the tensile strength, respectively, was demonstrated for the composites based on PP with hemp fillers in the amount of 20% and 30%. The use of 5% interfacial adhesion promoter (Scona 8112) indicates a positive effect on material adhesion, which ensures more effective mechanical properties. In the presence of a compatibilizer, the PP composites with 30% of the hemp filler showed better mechanical properties: the tensile strength is the same as that of pure PP, and the flexural strength is 24% higher.

Depending on the amount of hemp filler in the presence of the compatibilizer, the composites showed a significant increase in the tensile modulus during elongation (20–87%) and bending (17–58%).

The use of grass biomass fillers in the amount of 20 and 30% in composites based on polypropylene resulted in the reduction in tensile strength by 24 and 34%, respectively. The use of the 5% interfacial adhesion promoter (Scona 8112) improved the tensile strength of the composites by an average of 14%. However, the composites show a higher (13–33%) modulus of elasticity in relation to the pure PP. The use of grass fillers in the amount of 20 and 30% did not significantly affect the flexural strength of the composites. The modulus of elasticity determined during bending increased by almost 58% when the filler was used in the amount of 30%.

The effect of the different fillers from the halophyte biomass on both the tensile and flexural properties of the PLA composites are shown in Table 7 and Table 8.

The use of hemp fillers in the amount of 20 and 30% in composites based on PLA–Ingeo 3260HP resulted in a reduction in tensile strength by about 20 and 18%, respectively. The reduction in the flexural strength by 13–19% was demonstrated by the composites of PLA with hemp fillers. Depending on the amount of the hemp filler, the composites showed a significant increase in the modulus during elongation (57–91%) and bending (44–94%) in relation to pure PLA.

The use of grass biomass fillers in the amount of 20 and 30% in composites based on PLA–Ingeo 3260HP resulted in a reduction in tensile strength by about 24 and 36%, respectively. The composites show, respectively, an 11 and 17% increase in the modulus of elasticity in relation to pure PLA. The flexural strength of the composites decreased by about 28% for the 30% filler content. The modulus of elasticity determined during bending increased by 12 and 18% in relation to pure PLA.

It should be noted that the interaction between the matrix and the filler is an important factor influencing the mechanical properties. Despite the hydrophilic nature of the polylactide polymer matrix surface, similar to natural fillers, PLA composites with hemp and grass fillers do not show effective interface adhesion. The interfacial bonding strength of the natural fillers with the polymer matrix were lower, and at a lower value of the force acting on the samples, the fillers were detached from the matrix, and the entire load acted on the polymer matrix [37,38].

Moreover, it was found that the value of the reduction in tensile strength can be correlated with the content of natural fillers. For composites with 20% by weight of hemp filler, the reduction in tensile strength was 24.8% for PP composites and 20% for PLA composites, respectively. For PP composites with 30% by weight of hemp filler, the reduction in tensile strength was 38%. For composites containing 20 and 30% by weight of grass filler, the reduction in tensile strength was 24% and 34–36% for PP and PLA composites, respectively.

Summing up, it should be emphasised that the addition of a compatibilizer improved the adhesion at the interface of the PP composites with the hemp filler, which is confirmed by the better mechanical properties of composites in terms of elongation and bending.

## 3. Materials and Methods

### 3.1. Halophyte Biomass

The raw material used in the study was the biomass of grass—tall fescue and hemp of the Białobrzeskie variety from a saline field in the Kuyavian-Pomeranian Voivodeship (Poland), the average salinity of which, calculated as sodium chloride, was 3.5 g·L^−1^.

### 3.2. Bioethanol Production Process

#### 3.2.1. Halophyte Biomass Pretreatment

Grass and hemp biomass was subjected to preliminary crushing to particles of size 20–40 mm and dried at 50–55 °C for 24 h. Then, the material was disintegrated on knife mill SM-200 (Retsch, Hann, Germany) with a sieve of the mesh size of 2 mm.

The next step was the alkaline treatment of the halophyte biomass for 5 h with 1.5% sodium hydroxide at 90 °C [39]. NaOH/biomass weight ratio was 10:1. After the alkaline pretreatment was carried out, the biomass solution was filtered on a Büchner funnel, then washed with distilled water until neutralised, and dried in a laboratory dryer at 50 °C for 24 h. The alkali effect on the content of the released reducing sugar was determined using Miller’s method with 3,5-dinitrosalicylic acid (DNS) [40]. The raw material was incubated at 40 °C in 0.05 M citrate buffer pH 4.8 for 2 h using the enzyme preparation Flashzyme Plus 200 (AB Enzyme) at the dose of 20 FPU·g^−1^. The absorbance of the supernatant was measured at 530 nm on UV–VIS Spectrophotometer V-630, (Jasco, Pfungstadt, Germany).

#### 3.2.2. Enzyme Complex

In order to select the enzyme complex for the SSF process, tests were performed using selected enzymes—Flashzyme Plus 200 and Celluclast 1.5 L (Novozymes, Bagsværd, Denmark) and their supplementation with glucosidase 20 CBU·g^−1^ and xylanase 500 XU·g^−1^ (Sigma-Aldrich, Darmstadt, Germany).

The composition of Flashzyme Plus 200 (90 FPU·mL^−1^, 2430 XU·mL^−1^) is endoglucanase, cellobiohydrolase, cellobiase, xylanase and mannanase, and Celluclast 1.5 L (62 FPU·mL^−1^, 278 XU·mL^−1^) consists of cellulase from *Trichoderma reesei*.

Enzymatic tests were carried out for 5% of biomass with the enzyme in the amount of 10 FPU·g^−1^, at pH 4.8 and for 24 h at 38 °C. The selection criterion was the content of reducing sugars determined using the Miller’s method.

#### 3.2.3. Simultaneous Saccharification and Fermentation (SSF)

The SSF process was carried out in bioreactor Biostat B Plus (Sartorius, Goettingen, Germany) in 2-litre vessel equipped with pH, temperature, stirring and foaming controls. The temperature was maintained at 37 °C and stirred at 900 rpm; pH was controlled at 4.8 by adding 1 M NaOH or 1 M HCl. The mixture of Flashzyme Plus 200 and Celluclast 1.5 L enzymes in the amount of 10 FPU·g^−1^ was used for the hydrolysis process of biomass. The fermentation process was performed with the use of not hydrated, freeze-dried yeast *S. cerevisiae* at a dose of 1 g·L^−1^, which corresponded to cell concentration after inoculation of about 1 × 10^7^ cfu·mL^−1^. Duration of the SSF process was 24, 48 and 72 h.

Ethanol yield from 100 g of raw material *Y_s_* (g·100 g^−1^ of raw material) was calculated according to the following Equation (1) [41]:

(1) $$Y_{s}=\frac{Et\times 100}{M}$$

where *Et*—amount of ethanol in 1000 mL of tested sample (g); and *M*—mass of material weighed in 1000 mL fermentation sample (g).

### 3.3. Biocomposite Production Process

#### 3.3.1. Natural Fillers from Halophyte Biomass

Samples of dried halophyte biomass were grounded using a Rekord A (Jehmlich, Nossen, Germany) mill with a sieve separator with a mesh diameter of 1 mm. The sieve analysis of the natural fillers obtained in the milling process was performed using Analysette 3 Spartan (Fritsch, Idar-Oberstein, Germany) and their humidity were determined with moisture analyser MA.X2.A (Radwag, Radom, Poland).

#### 3.3.2. Polymer Matrix

The polymer matrix consisted of traditional thermoplastic polymer—polypropylene Moplen HP648T (Basell Orlen Polyolefins, Płock, Poland): density 0.9 g·mL^−1^ and mass flow rate (MFR) 53 g·10 min^−1^ (190 °C, 2.16 kg) and biodegradable polymer—poly(lactic acid) Ingeo 3260HP (NatureWorks, Blair, NE, USA): density 1.24 g·mL^−1^ and MFR 65 g·10 min^−1^ (210 °C, 2.16 kg).

To improve adhesion between the natural filler and the polypropylene matrix, the interfacial adhesion promoter Scona 8112 (S) (BYK-Chemie GmbH, Germany)—PP grafted with maleic anhydride—was used in an amount of 5% by weight.

#### 3.3.3. Preparation of Composites

Composites and biocomposites contained 20 and 30 wt% hemp (H) and grass (G) fillers were compounded with polypropylene (PP) and polylactide (PLA) in co-rotating twin screw extruder Leistritz MICRO 27 GL/GG-44D (Leistritz Extrusionstechnik, Nürnberg, Germany) with Brabender gravimetric feeding system (Brabender Technologie, Duisburg, Germany). Compounding parameters: barrel temperature profile 170–200 °C, extruder rotation speed of 150 rpm, throughput 16 kg·h^−1^.

Composites pellets were dried to achieve the appropriate process humidity. Polypropylene composites were dried in flow dryer EHD-25BT (Enmair Automation Machinery, Guangdong, China) at temperatures of 105 °C to a humidity level below 0.15%. Biocomposites based on biodegradable polymer were dried in molecular dehumidifier Drywell DW25/40 (Digicolor, Herford, Germany) at 70 °C (dew point −40 °C) to a humidity level below 0.02%.

Multipurpose test specimens—type A in accordance with ISO 3167 [36]—were moulded by hydraulic injection moulding machine Haitian Mars II Eco 600 kN (Haitian Plastics Machinery, Zhejiang, China). Barrel temperature profile: 180 °C (hopper), 185 °C, 190 °C and 190 °C (nozzle). Mould temperature was set at 40 °C.

### 3.4. Analytical and Testing Methods

The chemical composition of halophyte biomass before and after pretreatment was determined, i.e., cellulose acc. to TAPPI T17 m-55 [42], hemicellulose as the difference holocellulose acc. to TAPPI T9 m-54 [43] and cellulose, and lignin acc. to TAPPI T13 m-54 [44].

In order to provide a more complete picture of the molecular structure of grass and hemp biomass before and after the alkaline pretreatment, the analysis of FTIR spectroscopy was performed using a Fourier Transform Infrared Spectrometer ISS 66v/S (Bruker, Bremen, Germany) at wavenumbers of 400–4000 cm^−1^ [22].

The physical morphologies of halophyte biomass before and after the chemical treatment were performed by using Scanning Electron Microscope S-3400N (Hitachi, Japan) in high vacuum conditions. The samples were covered with gold dust.

The content of ethanol was determined using High Performance Liquid Chromatography on Elite LaChrom (Hitachi, Tokio, Japan) using an RI L-2490 detector, Rezex ROA 300 × 7.80 mm column (Phenomenex, Torrance, CA, USA), as the mobile phase used 0.005N H_2_SO_4_ at a flow rate of 0.6 mL·min^−1^, at 40 °C.

Composites’ tensile and flexural tests were carried out at room temperature with a universal testing machine Inspekt Table 50 (Hegewald & Peschke MPT, Nossen, Germany) as recommended by ISO 527 [45,46] and ISO 178 [47], respectively. A crosshead speed was set to 5 mm·min^−1^ in both tests. Tensile tests were performed using an MFA clip-on extensometer (Mess- & Feinwerktechnik, Velbert, Germany) with a nominal length of 50 mm.

### 3.5. Statistical Analysis

The experiments of ethanol fermentation were carried out in triplicates. Standard deviations were calculated using the analysis of variance ANOVA, Statistica 13.0 software (*p* < 0.05).

## 4. Conclusions

This study took on the challenge of broadening the knowledge and understanding of the halophyte species to achieve better use of the biomass from these plants. To sum, the biomass of both the tall fescue and the hemp of the Białobrzeskie variety from saline areas turned out to be a suitable source for the production of second-generation bioethanol and a natural filler for polymer composites based on traditional polymers (polypropylene) and biodegradable polymers (polylactide).

The SSF process made it possible to obtain an ethanol concentration for grass and hemp biomass at the level of approx. 7 g·L^−1^ (14 g·100 g^−1^ of raw material). In the case of composites, studies have shown that despite lowering the mechanical strength in terms of stretching and bending, it is possible to improve interfacial adhesion by modifying the compatibility between the phases of the natural filler and the polymer matrix.

This study will certainly have a positive impact on raising public opinion about the social and economic benefits of the bioproducts obtained from biomass sources using land unsuitable for agriculture.

## Figures and Tables

**Table 1:** Content of reducing sugars before (BP) and after (AP) sodium hydroxide treatment.

Halophyte	Sample	Reducing Sugars (mg·g^−1^)
Grass	BP	100.20 ± 0.09
	AP	354.59 ± 0.01
Hemp	BP	62.95 ± 0.10
	AP	187.95 ± 0.13

**Table 2:** Chemical composition of halophyte biomass (% of dry matter); BP: before pretreatment; AP: after pretreatment.

Halophyte	Sample	Cellulose (%)	Hemicellulose (%)	Lignin (%)
Grass	BP	33.69 ± 0.40	34.74 ± 0.39	17.08 ± 0.16
	AP	50.41 ± 0.18	25.23 ± 0.37	12.35 ± 0.07
Hemp	BP	47.34 ± 0.40	33.49 ± 0.68	13.94 ± 0.05
	AP	58.46 ± 0.29	22.12 ± 0.13	17.35 ± 0.26

**Table 3:** Content of reducing sugars after the enzymatic test.

Enzyme	Reducing Sugar (mg·g^−1^)	
	Hemp	Grass
Flashzyme Plus 200	338 ± 0.04	846 ± 1.00
Celluclast 1.5 L	342 ± 0.05	696 ± 0.52
Flashzyme/Celluclast 1.5 L (70/30)	420 ± 0.06	892 ± 0.02
Flashzyme/Celluclast 1.5 L (50/50)	430 ± 0.05	800 ± 0.44
Flashzyme/Celluclast 1.5 L (30/70)	355 ± 0.38	810 ± 0.34
Flashzyme/Celluclast 1.5 L (50/50)/β-glucosidase	351 ± 0.14	-
Flashzyme/Celluclast 1.5 L (50/50)/xylanase	324 ± 0.65	-
Flashzyme/Celluclast 1.5 L (50/50)/β-glucosidase/xylanase	343 ± 0.16	-
Flashzyme/Celluclast 1.5 L (70/30)/β-glucosidase	-	472 ± 3.29
Flashzyme/Celluclast 1.5 L (70/30)/xylanase	-	458 ± 1.81
Flashzyme/Celluclast 1.5 L (70/30)/β-glucosidase/xylanase	-	735 ± 1.86

**Table 4:** Particle size distribution and humidity of fillers from halophyte biomass.

Plant Biomass	Humidity (%)	Particle Size Distribution (%)						
		1 mm	0.5 mm	0.4 mm	0.25 mm	0.2 mm	0.1 mm	Below 0.1 mm
Grass	8.73	1.2	48.3	8.3	30.5	2.5	2.7	6.5
Hemp	7.65	1.1	53.8	14.7	15.8	8.5	2.6	3.5

**Table 5:** Tensile and flexular properties of PP/hemp composites.

Sample	Tensile Strength *δ*_M_ (MPa)	Tensile Modulus *E*_t_ (GPa)	Flexular Strength *δ*_fM_ (MPa)	Flexural Modulus *E*_f_ (GPa)
PP HP648T	31.0 ± 0.27	1.5 ± 0.02	41.5 ± 0.48	1.2 ± 0.13
PP-H20	23.3 ± 0.25	1.7 ± 0.04	39.5 ± 0.60	1.8 ± 0.17
PP-H20S5	27.5 ± 0.57	1.8 ± 0.05	40.7 ± 0.75	2.7 ± 0.16
PP-H30	19.3 ± 0.21	2.3 ± 0.03	37.0 ± 0.70	2.5 ± 0.22
PP-H30S5	31.4 ± 0.21	2.8 ± 0.05	51.6 ± 0.77	3.1 ± 0.14

**Table 6:** Tensile and flexular properties of PP/grass composites.

Sample	Tensile Strength *δ*_M_ (MPa)	Tensile Modulus *E*_t_ (GPa)	Flexular Strength *δ*_fM_ (MPa)	Flexural Modulus *E*_f_ (GPa)
PP HP648T	31.0 ± 0.27	1.5 ± 0.02	41.5 ± 0.48	1.2 ± 0.13
PP-G20	23.6 ± 0.19	1.7 ± 0.04	39.5 ± 0.27	1.3 ± 0.08
PP-G20S5	28.1 ± 0.25	1.8 ± 0.07	40.3 ± 0.35	1.4 ± 0.09
PP-G30	20.4 ± 0.14	2.0 ± 0.03	41.0 ± 0.40	1.9 ± 0.17
PP-G30S5	24.4 ± 0.27	2.0 ± 0.05	44.4 ± 0.27	1.9 ± 0.11

**Table 7:** Tensile and flexular properties of PLA/hemp composites.

Sample	Tensile Strength *δ*_M_ (MPa)	Tensile Modulus *E*_t_ (GPa)	Flexular Strength *δ*_fM_ (MPa)	Flexural Modulus *E*_f_ (GPa)
PLA 3260HP	64.5 ± 1.25	3.5 ± 0.07	108.6 ± 0.99	3.4 ± 0.14
PLA-H20	51.4 ± 0.99	5.5 ± 0.04	88.0 ± 2.37	4.9 ± 0.21
PLA-H30	53.0 ± 1.17	6.7 ± 0.05	94.7 ± 1.76	6.6 ± 0.18

**Table 8:** Tensile and flexular properties of PLA/grass composites.

Sample	Tensile Strength *δ*_M_ (MPa)	Tensile Modulus *E*_t_ (GPa)	Flexular Strength *δ*_fM_ (MPa)	Flexural Modulus *E*_f_ (GPa)
PLA 3260HP	64.5 ± 1.25	3.5 ± 0.07	108.6 ± 0.99	3.4 ± 0.14
PLA-G20	49.1 ± 0.30	3.9 ± 0.02	83.6 ± 0.43	3.8 ± 0.11
PLA-G30	41.2 ± 1.22	4.1 ± 0.02	78.1 ± 0.93	4.0 ± 0.20

## Data Availability

Not applicable.

## Sample Availability

Samples of the compounds are not available from the authors.
